# Reconstruction of micron resolution mouse brain surface from large-scale imaging dataset using resampling-based variational model

**DOI:** 10.1038/srep12782

**Published:** 2015-08-06

**Authors:** Jing Li, Tingwei Quan, Shiwei Li, Hang Zhou, Qingming Luo, Hui Gong, Shaoqun Zeng

**Affiliations:** 1 Britton Chance Center for Biomedical Photonics, Huazhong University of Science and Technology- Wuhan National Laboratory for Optoelectronics, Wuhan 430074, China; 2 Key Laboratory of Biomedical Photonics of Ministry of Education, Department of Biomedical Engineering, Huazhong University of Science and Technology, Wuhan 430074, China; 3 School of Mathematics and Statistics, Hubei University of Education, Wuhan 430205, China

## Abstract

Brain surface profile is essential for brain studies, including registration, segmentation of brain structure and drawing neuronal circuits. Recent advances in high-throughput imaging techniques enable imaging whole mouse brain at micron spatial resolution and provide a basis for more fine quantitative studies in neuroscience. However, reconstructing micron resolution brain surface from newly produced neuronal dataset still faces challenges. Most current methods apply global analysis, which are neither applicable to a large imaging dataset nor to a brain surface with an inhomogeneous signal intensity. Here, we proposed a resampling-based variational model for this purpose. In this model, the movement directions of the initial boundary elements are fixed, the final positions of the initial boundary elements that form the brain surface are determined by the local signal intensity. These features assure an effective reconstruction of the brain surface from a new brain dataset. Compared with conventional typical methods, such as level set based method and active contour method, our method significantly increases the recall and precision rates above 97% and is approximately hundreds-fold faster. We demonstrated a fast reconstruction at micron level of the whole brain surface from a large dataset of hundreds of GB in size within 6 hours.

Reconstruction of the mouse brain atlas at micron resolution is essential for the research of fine neural circuits. As a first step, brain registration^1^,^2^,^3^,^4^,^5^,^6^ requires a digital reconstruction of the brain surface from a large imaging dataset. Recent progress in microscopic imaging, including the sampling preparation^7^,^8^ and the microscopy imaging system^9^,^10^,^11^,^12^,^13^,^14^,^15^,^16^,^17^,^18^,^19^,^20^, have allowed imaging the whole mouse brain at sub-micron or micron spatial resolution and produced large-scale imaging datasets of mouse brains. These imaging datasets provide the basis for drawing the mouse brain atlas at micron resolution and, furthermore, are applicable to certain quantitative analyses in neuroscience, such as segmenting brain structure and digitizing neuronal morphology. Essentially, high-density sampling of the whole brain results in a very large data size, and the complexity of the whole brain dataset makes the quantitative analysis very challenging. Especially, digitizing micron resolution brain surface is also difficult, mainly because the brain dataset is large (hundreds of GB in size), and the signals at the brain surface are significantly variable.

Many methods have been proposed for digital reconstruction of the brain surface^21^,^22^,^23^,^24^,^25^,^26^,^27^,^28^,^29^,^30^, such as an improved geometric active contour model^21^ for brain surface extraction, which solved the boundary leakage problem and is less sensitive to intensity inhomogeneity to some extent; the deformable surface model^22^, which adapts the popular human brain extraction tool (BET) through the incorporation of information on the brain geometry and MR image characteristics of the rat brain; the deformable model^24^, which evolves to fit the brain’s surface by the application of a set of locally adaptive model forces; the locally linear representation-based classification method^25^ for brain extraction; and the brain extraction method^30^ using a hybrid level set based active contour neighborhood model. All of those methods are able to accurately reconstruct the brain surface, each for a specific purpose. However, these methods cannot be applied on a large-scale fluorescence imaging dataset of a mouse brain. On the one hand, these reconstruction methods are global methods. They are suitable for small data, such as MRI datasets (hundreds of MB size in 0.1 mm resolution); however, the computational efficiency of these methods makes applications in large brain datasets impractical. Although downsampling can reduce the data volume and make global methods feasible, the surface is not fine enough due to downsampling, which may reduce the resolution simultaneously. On the other hand, most of these methods are based either on the active contour model^21^,^22^,^23^,^24^ or on the level set method^30^; in principle, all of them require homogeneous signal intensity and are therefore unsuitable for the new imaging datasets. The reason is as follows: the active contour model^31^,^32^ reconstructs the shape of an object by minimizing the variational model that drives the initial surface elements move to the real surface. Because of a lack of a strong constraint in the movement direction of the initial surface elements, the path of the initial surface elements is seriously disrupted by the intensity inhomogeneity of the signals near the surface, resulting in a low-precision reconstruction. The level set based method^33^ is essential for using one or several optimal threshold values to distinguish between the foreground and background. Therefore, for the strong-intensity inhomogeneity of signals near the brain surface, one or several thresholds may meet difficulties in identifying the surface signals, and reconstructing the brain surface using the level set method may not be the optimal selection.

This obstacle propels us to establish a local method that does not lower the resolution and is not affected by the intensity inhomogeneity of the signals. Here, we proposed a resampling-based variational model (RSVM) for reconstructing the brain surface from a new imaging brain dataset. The resampling strategy makes our method local and does not lower the resolution; furthermore, fixes in the evolution direction can overcome the intensity inhomogeneity of the signals. In this model, the signals in the rays that are perpendicular to the initial brain boundary line form the resampling dataset. This model has two features: the initial boundary element must move along its corresponding ray; the final position of the initial boundary element is determined by the signals in its corresponding and adjacent rays, rather than one or several fixed thresholds. These two features make RSVM suitable for dealing with the surface signals with strong-intensity inhomogeneity. The results show that RSVM can effectively reconstruct the brain surface with recall and precision rates that are above 97% (manual reconstruction is regarded as the gold standard), which are obviously superior to the level set based method^33^ and the active contour methods^31^,^32^. The proposed model only analyzes the resampling dataset rather than the whole dataset and is suitable for addressing a large dataset. From the whole brain dataset (hundreds of GB in size), RSVM can reconstruct the brain surface within 6 hours, approximately 300-fold faster than the level set based method.

## Results

### The availability of RSVM

**Figure 1:**
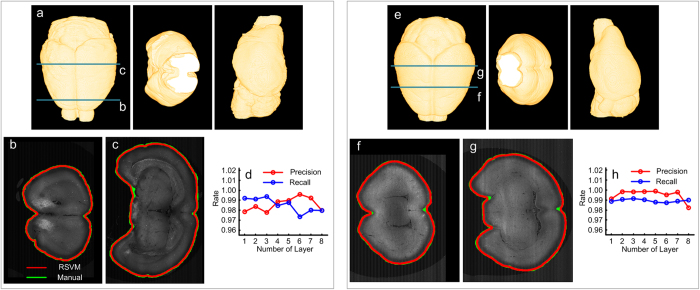
The effective reconstruction derived from RSVM. (**a**) The reconstructed brain surface using RSVM shown in different views: horizontal plane, coronal plane and sagittal plane, respectively. (**b**,**c**) A comparison of the reconstructed boundaries of two cross-sections (labeled by the dark green lines in (**a**)) derived from RSVM (red) and manually (green). (**d**) 8 cross-sections from the dataset in (**a**) were selected for a quantitative evaluation; the recall and precision rate are calculated by comparing the reconstructions derived from RSVM with the manual reconstructions. (**e**–**h**) The analysis of the other brain dataset reveals the same information as (**a**–**d**).

RSVM can effectively reconstruct the surface of a mouse brain. To verify this statement, two brain datasets produced by the 2p-fMOST imaging system^11^ were chosen. These two datasets contain 3478 × 4500 × 5000 and 4180 × 6000 × 4500 volume pixels, and the size of each pixel is 2 μm × 2 μm × 2 μm. Fig. 1a presents the horizontal plane, coronal plane and sagittal plane of the reconstructed surface of a mouse brain (from left to right) respectively, which are derived from RSVM to analyze one dataset. To display the reconstructions in detail, we extracted two cross-sections from the 3D mouse brain dataset, labeled by the dark green line in Fig. 1a. For each cross-section, the brain boundary lines reconstructed using RSVM (red) and manual (green) are provided, as shown in Fig. 1b,c. From a comparison of the reconstructed results derived from RSVM and manually, we conclude that RSVM can provide a high-precision reconstruction of the mouse brain surface. This conclusion has been further verified quantitatively, as shown in Fig. 1d. In a quantitative evaluation, the recall and precision rates are calculated. The recall rate is defined as the ratio of the area of the overlap region between the two regions enclosed by the boundary lines derived manually and by RSVM (briefly, called the overlap area) and the area of the region enclosed by the boundary line derived manually. The precision rate is defined as the ratio of the overlap area and the area of that derived from RSVM. In Fig. 1d, 8 cross-sections were selected from the dataset in Fig. 1a for a quantitative evaluation, and the recall and precision rates are all more than 97%. Fig. 1e–h that are from the analysis of the other brain dataset reveal the same information as Fig. 1a–d and further verify the effectiveness of RSVM in the reconstruction of a mouse brain surface.

### Resistance of signal interference during reconstruction

**Figure 2:**
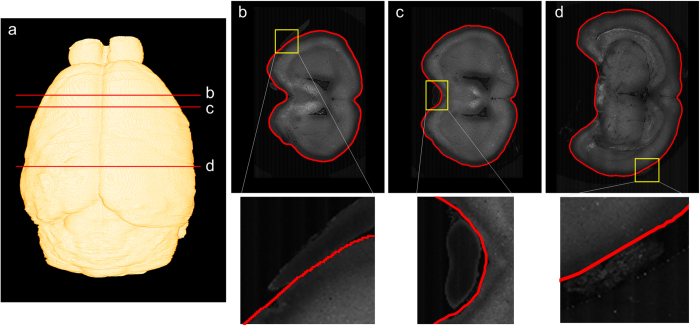
The resistance of signal interference during the reconstruction. (**a**) The reconstruction of the mouse brain surface derived from RSVM. (**b**–**d**) Three types of disturbance in the dataset are shown in the first row, and the enlargements of the yellow rectangle in the first row are shown in the second row.

RSVM is resistant to signal interference from the mouse brain boundary in reconstructing the mouse brain surface. To illustrate this point, we use the reconstruction results from the brain dataset with 3478 × 4500 × 5000 volume pixels. Using RSVM, the whole surface of the mouse brain was reconstructed and shown in Fig. 2a. From this reconstruction, three cross-sections labeled by the red lines in Fig. 2a were selected, and their reconstructed boundary lines (red curves) are shown in Fig. 2b–d. In Fig. 2b–d, the signals labeled by the yellow squares, which are useless and may be generated by some improper operation in sampling preparation, are very close to the boundary of a mouse brain and thus severely interfere with the brain surface reconstruction. However, RSVM can almost obtain perfect brain boundary lines of the cross-sections. This feature of RSVM can be revealed by the following two facts. On the one hand, similar to other variational models that have been used for reconstructing the boundary of an object, RSVM also requires a smoothness of the reconstructed boundary. The smoothness force reduces the influence of signal interference on the movement of the boundary elements. On the other hand, the design and direction of evolution in RSVM for each boundary element can be fixed, and the final position of the boundary element located at a predetermined region can be easily made. The predetermined region can also exclude the case in which a boundary element moves along a region of undesired signal.

### The robustness of RSVM to the sampling point density

**Figure 3:**
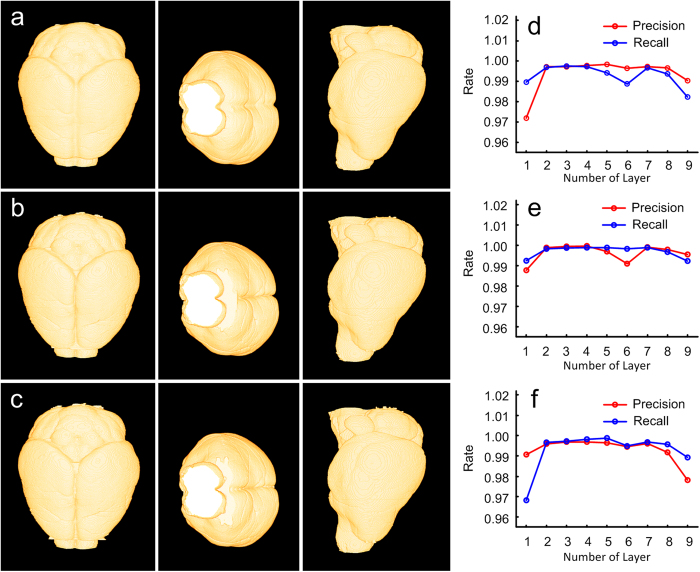
The stability of the sampling point density. (**a**–**c**) The reconstructions of the mouse brain surface derived from RSVM under different sampling point densities. The number of the sampling points is set to 360 (**a**), 540 (**b**) and 720 (**c**). (**d**) The recall and precision rates by comparing the reconstructions derived from RSVM shown in (**a**) and (**b**), in which (**b**) is regarded as the gold standard. (**e**) The recall and precision rates by comparing the reconstructions derived from RSVM shown in (**b**) and (**c**), in which (**c**) is regarded as the gold standard. (**f**) The recall and precision rates by comparing the reconstructions derived from RSVM shown in (**c**) and (**a**), in which (**a**) is regarded as the gold standard.

RSVM has a strong robustness to the sampling point density. Here, the sampling point density is measured by the number of discretized points on the initial boundary. Noting that RSVM produces an optimal boundary by minimizing the resampling-based variational model, and the initial boundary is necessary. We used the dataset shown in Fig. 1e for testing the robustness of RSVM to its direction of evolution in the sampling point density. In Fig. 3a,c, the reconstructed brain surfaces correspond to the initial boundaries, which are discretized into 360, 540 and 720 points. For each type of initial boundary, the corresponding reconstruction is displayed three ways, by the horizontal, coronal and sagittal plane (from left to right). Obviously, the difference between these reconstructions is indiscernible. We also quantified this difference. Compared with the reconstructions shown in Fig. 3a and Fig. 3b, we regarded the reconstruction in Fig. 3b as the gold standard and computed the recall and precision rates of the reconstructions in Fig. 3a. The result of the comparison is shown in Fig. 3d. Both of these reconstructed brain surfaces (Fig. 3a,b) contain 4500 cross-section boundary lines that were equally divided into 9 parts. In each part, every cross-sections boundary line from Fig. 3a and its gold standard was compared, and the recall and precision rates were generated. We use the average recall and precision rates for measuring the difference of this part reconstruction and the gold standard. Similarly, Fig. 3e reveals a comparison of the reconstructions in Fig. 3b,c under the assumption of the reconstruction in Fig. 3c, regarded as the gold standard, and Fig. 3f reveals a comparison of the reconstructions in Fig. 3c and Fig. 3a (gold standard). From Fig. 3d,f, we conclude that RSVM can obtain similar brain surfaces even if the sampling point density is set to a wide range.

### Comparison with the spherical-coordinated variational model

**Figure 4:**
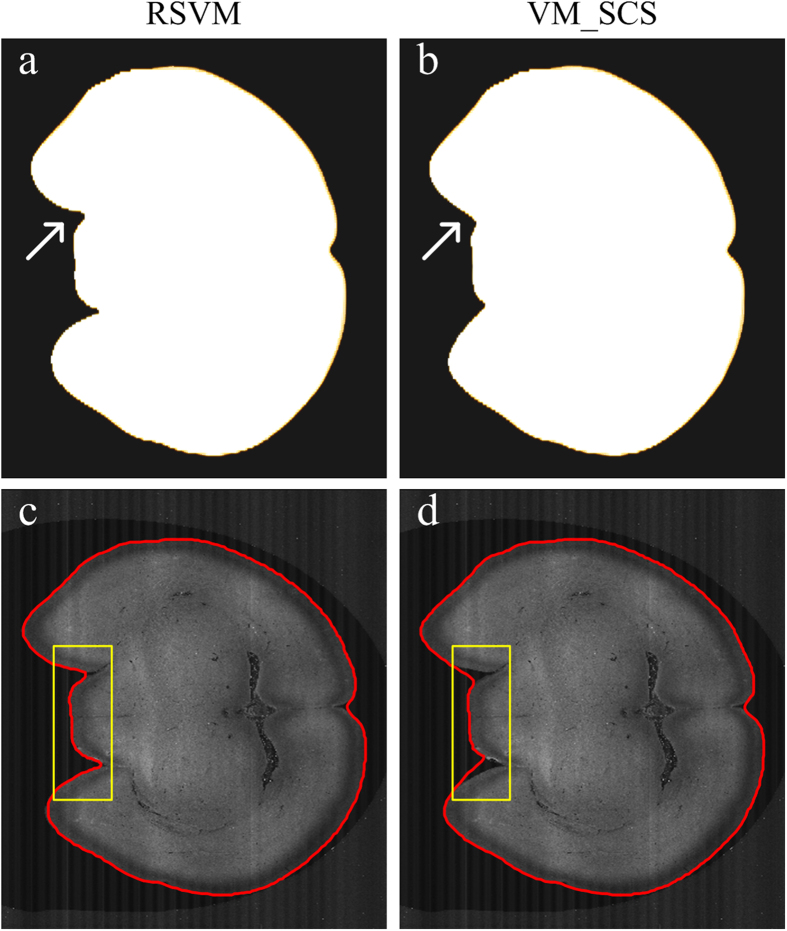
A comparison of the reconstructions derived from RSVM and the spherical-coordinated variational model (VM_SCS). (**a**–**b**) The reconstructions of 100 coronal planes derived from RSVM and VM_SCS, respectively. (**c**,**d**) The boundaries of a single coronal plane obtained through RSVM and VM_SCS, respectively. We can see the differences from the yellow rectangles in (**c**) and (**d**).

Compared with our previous variation model based on the sphere coordinate system (VM_SCS)^34^, RSVM can obtain the brain surface with a higher precision. Considering the huge time consumption of VM_SCS in the reconstructing of the whole brain surface, we selected a part of the whole brain dataset, which contains 4180 × 6000 × 100 volume pixels. For these 100 cross-sections, their boundary lines were derived from RSVM and VM_SCS, as shown in Fig. 4a,b. We conclude that RSVM has obvious advantages in driving the reconstructed boundary elements to reach the concave positions of real boundary lines (indicated by the arrows in Fig. 4a,b). This conclusion is clearly illustrated by a comparison of the reconstructions of a single cross-section (Fig. 4c,d).

### A comparison with other methods

**Figure 5:**
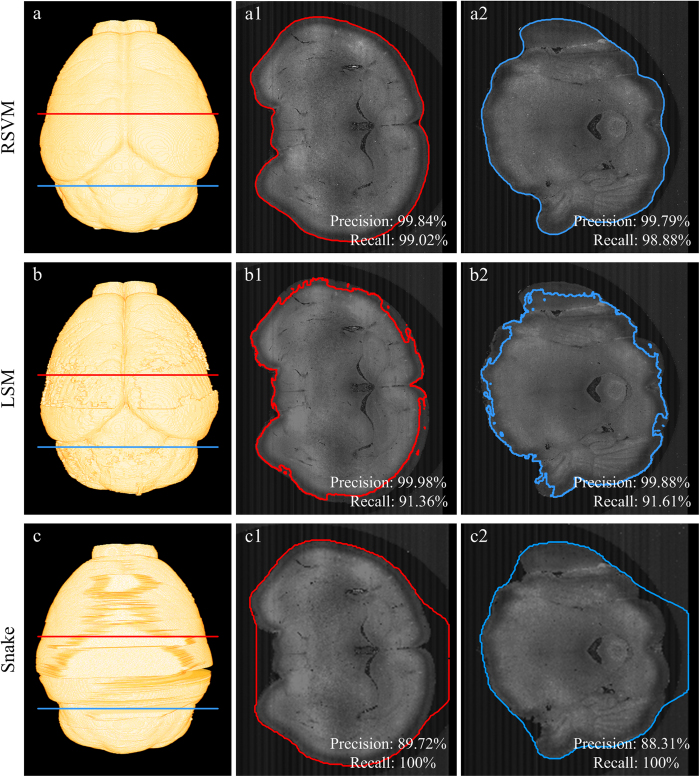
A comparison of the reconstructions derived from RSVM, the level set based method (LSM) and the active contour method (Snake). (**a–c**) The mouse brain surface reconstructed by RSVM, LSM and snake, respectively. (a1)–(c1) and (a2)–(c2) The boundaries of a single coronal plane obtained through RSVM, LSM and snake, respectively. (a1)–(c1) correspond to the red line in (**a–c**), and (a2)–(c2) correspond to the blue line in (**a–c**). The recall and precision rates are given on the according extracted cross-sections in (a1)–(c1) and (a2)–(c2).

Compared with the classical level set based method (LSM)^33^ and the active contour method (Snake)^32^, RSVM has a stronger ability to address boundary signals with a variable intensity. We used the dataset shown in Fig. 1e to verify this result. For each cross-section, reconstructing its boundary line will require approximately 700 seconds using LSM and approximately 2300 seconds using Snake; this speed is impractical for the reconstruction of a whole brain surface. Therefore, the size of the cross-section was decreased by merging 2 × 2 × 2 pixels into a single pixel for LSM and by merging 8 × 8 × 2 pixels into a single pixel for Snake; the rule used to merge multiple pixels into a single pixel (downsampling) is assigning the average pixel value of multiple pixels to the single pixel. From the dataset and two merging datasets, the brain surfaces derived from RSVM, LSM and Snake are shown in Fig. 5a,c, respectively. To display the comparison in detail, we extracted two cross-sections from the 3D mouse brain dataset, labeled by the red and dark green lines in Fig. 5a,c. The reconstructions derived from RSVM, LSM and Snake are shown in Fig. 5a1a,2, Fig. 5b1,b2 and Fig. 5c1,c2, respectively. We can see that RSVM produces the best brain boundary lines, which are close to their corresponding real boundaries. In a quantitative evaluation, the recall and precision rates are calculated, and all the frequencies of those methods are given on each extracted cross-sections.

Additionally, RSVM analyzes the resampling dataset rather than the whole dataset, and the time efficiency of RSVM is mostly determined by the size of the resampling dataset. For the classical level set based method (LSM) and active contour method (Snake), the whole dataset needs to be analyzed. Therefore, RSVM is faster than LSM and Snake in analyzing a large dataset. To illustrate this premise, 10 cross-sections from the brain datasets that all have a size of 4180 × 6000 pixels were selected. From the selected dataset, three groups of datasets were also generated by merging 2 × 2, 4 × 4, and 8 × 8 pixels into a single pixel. For each group of the dataset, the average time consumption of the reconstructions derived from RSVM, LSM and Snake were presented in Table 1. In analyzing the large size of the dataset (4180 × 6000 pixels), RSVM is 300-fold and 960-fold faster than LSM and Snake, respectively; for a relatively large size of the dataset (522 × 750 pixels), RSVM still has a 6-fold and 38-fold speed enhancement compared with LSM and Snake, respectively.

## Discussion

In this paper, we have proposed the RSVM method to reconstruct the brain surface from a new imaging brain dataset. The RSVM method is applicable to a large dataset and a brain surface with inhomogeneous signal intensity. The assumption of the RSVM method is that the mouse brain surface is smooth to some extent; this assumption is reflected in two aspects: first, there are no large changes between the adjacent layers; second, the boundary of each layer is smooth, i.e., a sharp change of the boundary is impossible when the position varies slowly. This is a normal and universal assumption for the mouse brain surface because the mouse brain surface is continuous and should not have large changes when the positions vary slowly. Therefore, the assumption is satisfied for other mouse brains. The limitation of the RSVM method is that we did not consider the situation of a shape’s separation and integration; therefore, RSVM cannot reconstruct a shape that is composed of multiple connected domains.

Similar to the spherical-coordinate-based variational model (VM_SCS)^34^, RSVM drives the boundary elements to move toward the real boundary under the combined action of the gradient force and the smoothness force. The convergent positions of the boundary elements correspond to the real boundary. It is clear that without an interference signal, the gradient force is dominant; on the contrary, when the interference signal is present, the smoothness force is dominant. Based on this premise, RSVM can reconstruct the brain surface under signal interference.

Different from the spherical coordinate based variational model (VM_SCS)^34^, RSVM can reconstruct the surface of nonconvex objects. In VM_SCS, the rays that the initial boundary elements move along are all started at the original point of the spherical coordinated system. This design constrains the diversity of the evolution of the initial boundary elements, causing a large loss in reconstructing a nonconvex shape. RSVM also has a requirement that the rays orthogonal to the initial boundary should be used to fix the evolution directions of the initial boundary elements. This requirement indicates RSVM can reconstruct any type of shape providing that the difference between the initial boundary and the real boundary is small. In fact, when RSVM obtains the boundary line from the cross-section, the boundary line is regarded as the initial boundary in the next reconstruction.

The level set based method (LSM)^33^ designs a variational model in the reconstruction and by minimizing the variational model, the optimal threshold can be obtained and used to distinguish between the foreground and background. Although the designed variational model requires a smoothness of the reconstructed boundary, a single threshold experiences difficulties in identifying the boundary with a variable signal-intensity. RSVM minimizes the variational model for obtaining the final positions of the initial boundary elements in the rays, and these positions form the reconstructed boundary line. The position of an initial boundary element is determined by the signals of the corresponding ray and its several adjacent positions in other rays, i.e., the threshold for identifying the boundary is variable. Therefore, RSVM is powerful for reconstructing a boundary line whose signal intensity is significantly changed.

Active contour methods (Snake)^31^,^32^ require an initial curve, and then, under the combined effect of internal force and external force, the initial curve moves toward the boundary of the shape. Generally speaking, the internal force is a representation of the smoothness of the shape boundary and is obtained by computing the average values. The external force of classical active contour methods is expressed as the gradient or the gradient vector flow (GVF) of the image. In active contour models, boundary elements move along the gradient or the gradient vector flow. However, when the signal near the brain surface is significantly changed, the gradient or the gradient vector flow is affected by the interference signal; therefore, the reconstruction is dependent on the initial curve, and the circumcircle of the boundary is used as the initial curve of Snake. While in the RSVM method, the boundary element moves along its ray direction, this design allows the RSVM method to overcome the situation of a significantly variable signal near the brain surface.

To address the high-throughput image stacks, computational efficiency is an essential factor. RSVM only analyzes the resampling dataset rather than the whole dataset; the resampling strategy makes our method local and does not lower the resolution and is suitable for addressing a large dataset. From the whole brain dataset (hundreds of GB in size), RSVM can reconstruct the mouse brain surface within 6 hours in an Inter(R)Xeon(R)CPU 3.10 GHz computing platform.

## Methods

### The resampling-based variational model for the reconstruction of a brain surface

**Figure 6:**
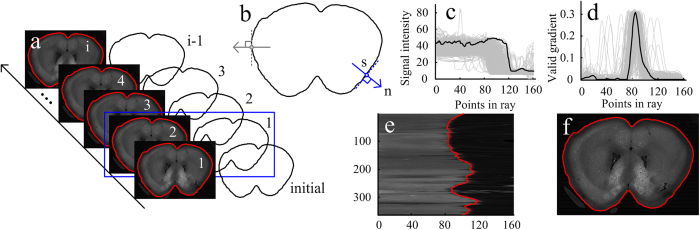
A flow chart of RSVM. (**a**) In the boundary reconstruction of the first coronal plane (z = 1), the initial boundary is given manually. The reconstructed boundary of the coronal plane (z = 1), regarded as the initial boundary, is used for reconstructing the boundary of the coronal plane (z = 2). By repeating this procedure, a series of reconstructed boundaries that form the brain surface are produced. (**b**) Generation of the resampling dataset. The variable *s* represents the sequence number of the discretized initial boundary element, and the variable *n* represents the exterior normal direction. (**c**) The signals in rays obtained via resampling. (**d**) The valid gradient signals calculated from the curves in (**c**). The black curves in (**c**) and (**d**) serve as the examples. (**e**) From the signals in (**c**), the curve boundaries in the resampling dataset were calculated using RSVM. (**f**) The curve boundaries in the resampling dataset were converted to the boundary corresponding to the coronal plane image.

The position of a voxel in brain image stacks is labeled by the coordinate (x, y, z) in an orthogonal coordinate system. The x-y plane of the imaging dataset is briefly called the coronal plane, and the boundaries of a series of coronal planes associated with the z direction form the brain surface. The boundary of a coronal plane can be discretized and expressed by a group of nose-to-tail points. To acquire the boundary of a coronal plane, the initial boundary needs to be pre-provided. In the boundary reconstruction of the first coronal plane (z = 1), the initial boundary is given manually. The reconstructed boundary of the coronal plane (z = 1), regarded as the initial boundary, is used for reconstructing the boundary of the coronal plane (z = 2). By repeating this procedure, a series of reconstructed boundaries that form the brain surface are produced, as shown in Fig. 6a. Considering the continuous property of the brain surface, the boundary differences between the two adjacent coronal planes are subtle, and this setting of the initial boundary is reasonable.

After ascertaining the initial boundary, we designed a resampling-based variational model that can drive the initial boundary to evolve to the real boundary. For the given initial boundary element denoted by *s*, we assume that its movement directions should be perpendicular to the initial boundary, i.e. the exterior or interior normal direction. According to this assumption, the position of *s* can be denoted by *r*_0_ + *r*(*s*)*n. Here, *r*_0_ is the initial position of the element *s*, *n* is set to the exterior normal direction, and *r*(*s*) is an unknown scale. When *r*(*s*) changes in a given interval, the set of the corresponding positions is called the ray determined by *s*. The notes of *s* and *n* are also further illustrated in Fig. 6b. The real boundary can be modeled as the function *r*(*s*) when the initial boundary is set. To resolve *r*(*s*), we assumed that the boundary of the coronal plane is smooth to some extent, i.e., a sharp change of *r* is impossible when *s* varies slowly; and that the intensity of the differential signal on the boundary is stronger than that in the inside and outside of the coronal plane. Based on these two assumptions, we design and minimize the following variational model for resolving *r*(*s*), written as


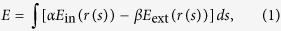


Here, E_in_(*r*(*s*)) and E_ext_(*r*(*s*)) are functions with respect to *r*(*s*). E_in_ is the internal force that represents the smoothness of the boundary, i.e., the distance between the adjacent boundary elements cannot be too large, which allows our method to overcome the situation of a significantly changed signal near the brain surface; E_ext_ is the external force, which represents a valid gradient of resampling rays and makes the initial boundary elements move toward the real boundary. Under the combined effect of the internal force (E_in_) and external force (E_ext_), we can reconstruct the boundary of a coronal plane. The small integration value of E_in_(*r*(*s*)) indicates that the function *r*(*s*) is slowly changed when *s* varies, corresponding to the smoothness of the boundary. Maximizing the integration value of E_ext_(*r*(*s*)) will drive the element *s* to move to the boundary position where the intensity of the differential signal is the strongest. Therefore, in the parameters setting, a large value of *α* and a small value *β* are much more likely to stress the smoothness of *r*(*s*) than to stress the detailed reconstructions. Conversely, the more detailed reconstructions can be obtained. From the above analysis, E_in_(*r*(*s*)) and E_ext_(*r*(*s*)) are designed as


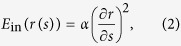



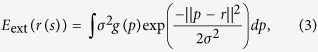


Here, *g*(*p*) denotes the differential signal of the position *r*_0_ + p*n, in which *r*_0_ is the initial position of *s*, and *p* is perpendicular to the initial boundary and determined by *r*_0_. *σ* is the parameter used to measure the difference between *p*(*s*) and *r*(*s*). Obviously, finding the peak position of the function *g*(*p*) is equivalent to maximizing E_ext_(*r*(*s*)), and using the optimal r* from maximizing E_ext_(*r*(*s*)), the final position of *s* can be determined by *r*_0_ + r*n and regarded as the reconstructed boundary position.

### The numerical solution of the designed variational model

To resolve *r*(*s*) from the variational model (1), we introduce the procedure used in the active contour method^31^,^32^ or the solving spherical-coordinated variational model^34^. It is well known that if *r*(*s*) is the optimal solution that minimizes the variational model (1), *r*(*s*) must satisfy the Euler equation


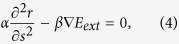


To solve the equation (4), we introduce a variable about time *t* and treat *r*(*s*) as a function of *s* and time *t*. The new equation about *r*(*s*, *t*) is established as
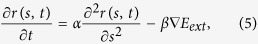


In fact, when *r*(*s*, *t*) stabilizes, i.e., the right hand side of the equation (5) is near zero, Equation (5) approaches equation (4). Equation (5) can be given as


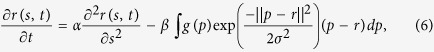


when equation (3) is substituted into equation (5). To obtain the stable *r*(*s*, *t*), we discretize the equation (6), and the corresponding differential equation is given as
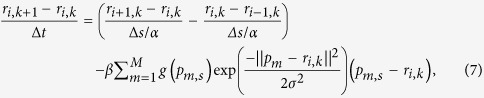


In which *r*_i, k_ is an abbreviation of *r*(*s*_i_, *t*_k_), *t*_k_ represents sampling points in the time interval, *s*_i_ represents the boundary element, and *p*_m, s_ is the sampling point in the ray determined by *s*_i_. For simplifying equation (7), we set Δs/α to one and obtain the iterative scheme


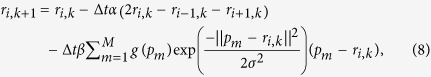


Using equation (8) for iteratively computing *r*_i, k+1_ until convergence, we can achieve the numerical solution of *r*(*s*).

### The processing of the extraction of the boundary of the coronal plane

This section illustrates how to obtain the numerical solution of *r*(*s*) and contains three parts. Part one introduces the generation of the resampling dataset, and Part two describes the procedure for the computation of the valid gradient signal, i.e., *g*(*p*_m_). In Part three, the process of the reconstruction of the brain boundary line is summarized, and the setting of some parameters for the reconstruction is also provided.

#### Step 1

Generation of the resampling dataset. As previously stated, the initial boundary determining the resampling dataset is necessary for reconstructing the brain boundary line. First, by discretizing the initial boundary, a group of nose-to-tail positions denoted by *r*_01_, *r*_02_, …, *r*_0n_, are generated. For each position *r*_0i_ on the initial boundary, the position of its corresponding boundary element *s*_i_ can be denoted by *r*_0i_ + *r*(*s*_i_)*n, with *n* being the exterior normal direction of the initial boundary at the position *r*_0i_, and *r*(*s*_i_) being a scale value. To simplify the calculation, we used the exterior normal direction of the straight line *r*_0i−1_*r*_0i+1_ to take the place of the exterior normal direction of the initial boundary at the position *r*_0i_, i.e., *n* is calculated by the exterior normal direction of the straight line *r*_0i−1_*r*_0i+1_. When *r*(*s*_i_) changes, the corresponding positions *r*_0i_ + *r*(*s*_i_)*n form a position set called the ray determined by *s*_i_ and *n*. Second, we set *r*(*s*_i_) to the integers in the interval [–M, M] for obtaining the position *p*_*m, s*_ (m = –M, –M + 1,..,M) on the ray determined by *s*_i_ and calculate the signal of the position *p*_*m, s*_, denoted by *v*(*p*_m, s_), by averaging the values of the pixel that the position *p*_m, s_ is in and the 25-connected pixels of the position *p*_m, s_. Note that the signals of *p*_m, s_ are shown in Fig. 6c, an appropriate value of M makes the boundary of the coronal plane fully included in the resampling region. Finally, for all the rays, their corresponding positions and signals form the resampling dataset are denoted by the set


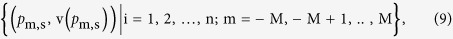


#### Step 2

The calculation of the valid gradient signal in the resampling dataset. To calculate the valid gradient signal, i.e., *g*(*p*_m, s_) in equation (8), we describe the procedure that contains the following steps.

(a) The modification of the signal of *v*(*p*_m, s_) in the resampling dataset (9). For eliminating the extreme values of the signal, the value of *v*(*p*_m, s_) is constrained to the interval [L_1_, L_2_], where L_1_ and L_2_ are the average of *v*(*p*_m, s_) for m = 1,2, …, M and for m = –M,–M + 1,…,−1, respectively, i.e.,


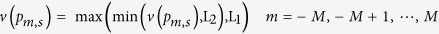


_In which L1 and L2 are fixed when the image is given, and this operation makes *v*(*p*_m, s_) lessen the interference from a sudden change signal. Note that when m is negative, the position *p*_m, s_ is in the region enclosed by the initial boundary; conversely, *p*m, s is outside of the enclosed region. In our images, the signal intensity in the enclosed region is generally more than that outside of the enclosed region, indicating that L1 is far less than L2._

(b) Smoothness of the modified signals. Using the averaging method to smooth the modified signal *v*(*p*_m, s_). Every signal *v*(*p*_m, s_) for m = –M,–M + 1,…,M is obtained by averaging *v*(*p*_m−1, s_), *v*(*p*_m, s_) and *v*(*p*_m+1, s_) and for 10 times.

Generation of the valid gradient signal. For the smoothed signal *v*(), we compute its differential signal by *ν*(*p*_m, s_) = *ν*(*p*_m+1, s_)–*ν*(*p*_m, s_), m = –M,–M + 1,…,M − 1, and the valid gradient signal is given as


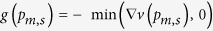


This setting is based on following facts: minimalizing the variational model (1) requires that the signal value of *g*(*p*_m, s_) is more than zero and as large as possible when *p*_m, s_ is on the real boundary, and the signal of the position inside the boundary is more than that outside the boundary, indicating that the value of the differential signal on the boundary is less than zero. Therefore, we take the converse of the differential signal. In addition, from the above analysis, we conclude that the nonnegative values of the differential signal are useless for the minimalizing variational model (1), and thus we set these nonnegative values to zeroes. Note that the signals of *g*(*p*_m, s_) are shown in Fig. 6d.

#### Step 3

Brain surface reconstruction. For all the initial boundary elements, the initial corresponding scale *r*(*s*_i_) are all set to zeros, and by repeatedly calculating the equation (8), the convergent values of these scales can be ascertained. Using these convergent values, we can calculate the final positions of these boundary elements using the expression in section *Step 1*. These final positions form the reconstructed boundary of the given coronal plane associated with the z-direction, and these reconstructed boundaries, arrayed along with the z-direction, form a reconstructed brain surface, as shown in Fig. 6a,e. In equation (8), there are some parameters that need to be predetermined. We set the parameter *β* to


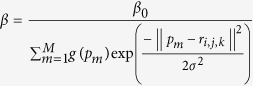


With σ being 0.5 and *β0* being 0.2, and set Δt and α to 1 and 0.8, respectively.

RSVM reconstructed the mouse brain surface of two datasets in Fig. 6a,e by giving two initial boundary lines.

### Sample preparation

All the experiments were performed in accordance with the guidelines of the Experimental Animal Ethics Committee at Huazhong University of Science and Technology. All the experimental protocols were approved by the Institutional Animal Ethics Committee of Huazhong University of Science and Technology. Thy1-EGFP M line transgenic mice were used in this study. The sample-preparation procedures were as follows: 1) deeply anesthetized the mice; 2) intracardial perfusion; 3) extracted the entire brain and post-fixed it; 4) rinsed the brain; 5) dehydrated the brain; 6) infiltrated the entire brain; and 7) embedded the brain in gelatin capsules filled with pre-polymerized GMA and polymerized it. A detailed description of the parameters, such as the solutions and its concentrations and the timing and frequency in every sample preparation procedure is provided elsewhere^8^,^10^.

### Imaging system

The imaging system, called two-photon fluorescence micro-optical sectioning tomography (2p-fMOST)^11^, can be used to section and image the entire embedded mouse brains and to obtain the large-scale imaging datasets. All the image tiles of every coronal plane were recorded in a folder automatically, and then through image pre-processing procedures^35^, the raw image tiles were montaged into the coronal plane sequence images. This step removed the inhomogeneous illumination pattern and redundant portions at the same time. The original size of the volume pixel is 0.5 μm × 0.5 μm × 2 μm. The total amount of uncompressed volume of each brain was approximately 1 TB and contained approximately 5000 coronal sections. To balance the resolution of the different directions, the size of the cross-section was decreased by merging 4 × 4 pixels into a single pixel, and the original brain dataset was converted to the testing dataset with the size of the volume pixel of 2 μm × 2 μm × 2 μm.

## Additional Information

**How to cite this article**: Li, J. *et al.* Reconstruction of micron resolution mouse brain surface from large-scale imaging dataset using resampling-based variational model. *Sci. Rep.* **5**, 12782; doi: 10.1038/srep12782 (2015).

## Figures and Tables

**Table 1:** A contrast of the computation time among RSVM, LSM and Snake.

Pixel binning	Size (pixels)	RSVM (s)	LSM (s)	Snake (s)
1 × 1	4180 × 6000	2.42	745.65	2334.88
2 × 2	2090 × 3000	2.02	188.01	530.89
4 × 4	1045 × 1500	1.87	44.57	281.04
8 × 8	522 × 750	1.88	12.13	71.99

The 1st column is the pixel binning mode. The 2nd column is the size (pixels) of the image after pixel binning. The 3rd column is the computation time to calculate the image with the size in the 2nd column using RSVM. The 4th column is the computation time to calculate the image with the size in the 2nd column using LSM. The 5th column is the computation time to calculate the image with the size in the 2nd column using Snake. The units of time in the 3rd to 5th columns are second (s).
